# The renaissance of organic radical chemistry – deja vu all over again

**DOI:** 10.3762/bjoc.9.312

**Published:** 2013-12-04

**Authors:** Corey R J Stephenson, Armido Studer, Dennis P Curran

**Affiliations:** 1 Department of Chemistry, University of Michigan, Ann Arbor, MI 48109, USA; 2 Institute of Organic Chemistry, Westfälische Wilhelms-University Münster, 48149 Münster, Germany; 3 Department of Chemistry, University of Pittsburgh, Pittsburgh, PA, 15208, USA

**Keywords:** free radical

Long-thriving movements often have a habit of continually reinventing themselves in a process called renaissance. The word itself is an English reinvention (some would say theft) of the French word for “rebirth”. Renaissances often occur in waves with each new wave typically building on the prior one. The European Renaissance is a perfect example, occurring in waves over about three centuries. Quoting from Yogi Berra, after a few of these waves that bear certain resemblances, it starts to feel like “deja vu all over again” [1].

The discipline of organic radical chemistry dates back over 110 years, and it has been thriving for decades because it continuously reinvents itself. Often this happens when groups of researchers with different interests and expertise immigrate to the field.

About 30 years ago there began a flourishing period in the field of organic radical chemistry that delivered groundbreaking results, especially in organic synthesis. By the 1990’s, radical reactions (especially cyclizations) were broadly recognized as powerful tools to make molecules. This Renaissance I in organic radical chemistry was built on prior renaissances in polymer and physical (especially physical organic) chemistry of radicals. In turn, the knowledge gained during Renaissance I was diverted to spark new waves of developments in neighboring disciplines such as biology and materials science. A comprehensive overview of the various aspects of modern radical chemistry addressing all these major fields can be found in the recently published four-volume “Encyclopedia of Radicals in Chemistry, Biology and Materials” [2].

Over the past few years, the process has again come full circle as research in organic radical chemistry, especially synthetic radical chemistry, has been reignited. This renaissance, Renaissance II if you will, is documented by the many exciting contributions recently published in high impact journals. This Thematic Series on radical chemistry in the Beilstein Journal of Organic Chemistry brings together papers from top research groups and representing many of the most significant themes in the current field of radical chemistry.

A partial list of topics that have seen new developments in the past few years follows. Many of these themes are represented once or multiple times in papers of the Thematic Series:

a) Base mediated homolytic aromatic substitution (BHAS) [3],

b) photoredox catalysis [4–8],

c) redox chemistry using Bu_4_NI in combination with *t*-BuOOH [9],

d) transition metal catalyzed processes where radicals are suggested to interact directly with copper, nickel, zinc, palladium, gold and so on [10–16],

e) radical trifluoromethylation [17] and radical fluorination [18–20],

f) natural product synthesis [21],

g) new main group radical chemistry involving elements like boron [22], phosphorous [23] and selenium [24], tellurium [25], among others,

h) synthesis or functionalization of all sorts of heterocyclic ring systems, especially heteroaromatic systems [26–27],

i) radical chemistry with water or in water [28],

k) sequential transformations with both reductants and oxidants,

l) radical chemistry in materials science [29–30], and

m) high level computations of radicals and their reactions [31].

Just as in Renaissance I, the field of radical chemistry is currently being joined by all sorts of researchers whose primary interests have until recently been elsewhere. In the 1980’s there were immigrants from natural products chemistry and synthetic methodology. Today, many of the new immigrants again come from a background of synthesis, often with a focus on catalysis. They bring along new ideas and different perspectives. As both new and established researchers have dared to try radical reactions in challenging new areas, their results have lit up the field.

Successful explorers of all ages have always taken the best existing maps on their voyages of discovery. These maps were invaluable, even though they inevitably were incomplete and even inconsistent. Little by little, the inconsistencies got fixed and the empty spaces got filled in. The reviews in the Encyclopedia of Radical Chemistry and other sources serve as maps to drive organic radical research in new directions. Like old maps of the earth, these maps are also inevitably incomplete and at times inconsistent. But they are powerful aids to help you understand both where you are and where you are going. Or, you can forget the maps and wander out on your own. But to again quote Yogi, “if you don’t understand where you are going, you might end up someplace else [1].” To all those new folks joining the field, we welcome you to the world of organic radical chemistry. Get yourself a good set of maps and start exploring.

There is one final parallel to draw between Renaissance I and Renaissance II in organic radical chemistry. In 1985, a thematic special issue on organic radical chemistry in Tetrahedron organized by Bernd Giese [32] brought many of the advances of radical chemistry to a wider audience. Similarly, this Thematic Series edited by Corey Stephenson advances the same goal.

Take some time to read a few of the papers in this Thematic Series. Think of the papers as evolving maps of regions of radical chemistry under exploration. Allow yourself to be drawn into the exciting world that is modern organic radical chemistry.
